# Expression of Reg IV and SOX9 and their correlation in human gastric cancer

**DOI:** 10.1186/s12885-018-4285-x

**Published:** 2018-03-27

**Authors:** Na Zhang, Dandan Chai, Huifen Du, Kesheng Li, Wenguang Xie, Xingwen Li, Rong Yang, Xiaowen Lian, Yang Xu

**Affiliations:** 1 Department of Medicine Biotechnology, Medicine and Science Research Institute of Gansu Province, Lanzhou, China; 2 College of Life Sciences, Lanzhou University, Lanzhou, China; 3 Department of Surgery, Tumor Hospital of Gansu Province, Lanzhou, China; 4 Department of pathology, Tumor Hospital of Gansu Province, Lanzhou, China

**Keywords:** Reg IV, SOX9, Correlation, Gastric cancer

## Abstract

**Background:**

Reg IV is a member of the regenerating gene family and has been demonstrated to be overexpressed in gastric cancer. However, the functional mechanism of Reg IV in gastric cancer is still unclear.

**Methods:**

Expression of Reg IV and SOX9 were investigated by immunohistochemistry (IHC) and real-time PCR, and the correlation between the expression of Reg IV and SOX9 was analyzed in gastric cancer tissues. Reg IV expression vectors and a siRNA of Reg IV and SOX9 were transfected into human gastric cancer cells and the protein and mRNA levels of Reg IV and SOX9 were investigated by western blot and real-time PCR. The invasion and migration ability of gastric cancer cells with overexpressed Reg IV and with gene silence of Reg IV and SOX9 were examined by transwell chambers and wound healing assay.

**Results:**

The Reg IV and SOX9 protein expression levels were both significantly higher in gastric cancer tissues compared with adjacent tissues (*p* = 0.022, *p* = 0.003). Reg IV protein expression significantly correlated with tumor invasion depth (*p* <  0.001), but had no significant correlations with age, clinical stage or lymph node metastasis. SOX9 protein expression also had no significant correlations with age, clinical stage, tumor invasion depth or lymph node metastasis. Reg IV transcript expression demonstrated a significant correlation with invasion depth and lymph node metastasis (*p* = 0.005, *p* <  0.001) and no significant correlations with age, clinical stage, tumor tissue differentiation or tumor size. SOX9 transcript expression demonstrated a significant correlation with invasion depth and tumor tissue differentiation (*p* = 0.044, *p* = 0.007) and no significant correlations with age, clinical stage or tumor size. The Reg IV expression showed a positive correlation with the SOX9 expression (*p* <  0.000, *p* = 0.008). Overexpression of Reg IV could upregulate SOX9 expression and promote invasiveness and migration of tumor cells, and silencing of Reg IV could downregulate SOX9 and inhibit invasiveness and migration of tumor cells in MKN-45 and AGS cells. On the other hand, silencing of SOX9 could upregulate Reg IV protein expression.

**Conclusions:**

Our study demonstrated that Reg IV positively regulates the expression of SOX9 and is involved in tumor cell invasion and migration in gastric cancer.

**Electronic supplementary material:**

The online version of this article (10.1186/s12885-018-4285-x) contains supplementary material, which is available to authorized users.

## Background

Gastric cancer, known for its high incidence and mortality rate, is the fourth most common malignancy in the world [1]. Invasion and metastasis of primary tumors contributes to the overwhelming mortality, but the underlying mechanism remains poorly understood [2, 3]. Metastasis is acknowledged to be a multistep process sequentially involving cell motility, tissue invasion and endosmosis, dissemination, and proliferation to form a distant metastasis tumor [4, 5]. Although studies on cancers have revealed various genes and gene products that regulate this malignant process [5], the molecular pathway of invasion and migration in gastric cancer need to be further elucidated.

Human regenerating islet-derived gene 4, known as Reg IV, is a newly-discovered member of the calcium-dependent lectin superfamily, and was characterized as an upregulated gene in inflammatory bowel diseases in 2001 [6]. Reg IV was found to be highly expressed in various cancerous tissues, such as gastric cancer [7, 8], colorectal cancer [9, 10], pancreatic cancer [11, 12] and prostate cancer [13], compared to the adjacent normal tissues. Reg IV enhances invasion in pancreatic cancer by upregulating MMP-7 and MMP-9 [11]. A previous study revealed that the invasion and migration abilities significantly increased after transfecting the carbohydrate-recognition domain (CRD) of Reg IV into LoVo colorectal cancer cells (CRD-deficiency) compared with empty controls and untreated LoVo cells [14]. The results indicated that Reg IV accelerated colorectal cancer cell migration and invasion via its CRD. It has been reported that *REG4* plays a multifunctional role of intestinal morphogenesis, motility, and invasion in the development and progression of colon cancer [15]. In addition, Reg IV increased invasion capacities and inhibited cell apoptosis by activating the EGFR/Akt/AP-1 signaling pathway in colon cancer [16]. In gastric cancer, Reg IV enhances peritoneal metastasis and inhibits apoptosis through upregulation of the level of several anti-apoptosis factors: Bcl-2, Bcl-XL, survivin, phosphorylated Akt, and phosphorylated EGFR; and deregulation of nitric oxide and 5-FU induced apoptosis [17, 18]. Other research also found that Reg IV promotes growth, proliferation, and migration in MKN-45 gastric cancer cells through the protein kinase B (Akt) pathway [19]. SOX9 (SRY related high-mobility group box 9), a transcriptional regulator that is essential to chondrogenesis and the formation of the male gonad [20, 21], has been found to activate Akt expression in pancreatic ductal adenocarcinoma [22]. Another previous study demonstrated that EGFR induced SOX9 through ERK1/2 signaling to support epithelial migration and wound repair in urothelial neoplasms [23]. Moreover, SOX9 was identified as one of the downstream targets of Reg IV on GeneChip analysis in gastric cancer [24]. Based on the above studies, we speculated that Reg IV and SOX9 may have certain correlations in their contributions to the development and progression process of gastric cancer. Despite the above advances, the functional mechanisms for the effects of Reg IV and SOX9 in human gastric cancer remain unknown.

In this study, we revealed that Reg IV and SOX9 were both overexpressed in human gastric cancer tissues, and the Reg IV transcript and protein expression demonstrated a positive correlation with the SOX9 transcript and protein expression. In addition, we investigated the role of Reg IV in regulating SOX9 in MKN-45 and AGS cells. The results showed that Reg IV potentiated invasion and migration by modulating SOX9 expression, and there was a feedback effect between Reg IV and SOX9 in gastric cancer cells. The results of this study will be helpful to understand the mechanism by which Reg IV promotes gastric cancer invasion, and may provide useful information for the clinical diagnosis and treatment of gastric cancer.

## Methods

### Tissues

Primary gastric adenocarcinoma tissues, diagnosed by clinical and histopathological evidence, were obtained from 195 patients undergoing surgery at Tumor Hospital of Gansu Province between March 2014 and April 2015. Samples of corresponding adjacent normal tissues were collected over 5 cm from the primary focus at the same time. No patients received chemotherapy or radiotherapy before surgery. Formalin-fixed, paraffin-embedded tissue specimens from 102 cases of gastric cancer and 40 cases of adjacent tissues were prepared by the pathology department for immunohistochemistry (IHC). The other 93 cases and paired adjacent tissues were used for real-time PCR and were immediately snap-frozen in liquid nitrogen and stored at − 80 °C until RNA extraction. The clinicopathological data, including age, gender, tumor size, and tumor-node-metastasis (TNM), were obtained from clinical and pathologic records. Table 1 lists the characteristics of patients registered in this study.

**Table 1:** Patient characteristics

Clinicopathologic features	IHC staining	Real-time PCR	PR (%)	Total
Case no.	102	93		195
Age(y)				
< 60	61	48	55.9	109
≥60	41	45	44.1	86
Gender				
Man	76	70	74.9	146
Female	26	23	25.1	49
Tumor size(cm)				
< 5	57	58	59.0	115
≥5	45	35	41.0	80
Tissue differentiation				
Well	5	1	3.1	6
Moderately	56	56	57.4	112
Poorly	41	36	39.5	77
Depth of invasion				
T1 + T2	27	22	25.1	49
T3 + T4	75	71	74.9	146
Lymph node metastasis				
No	32	22	27.7	54
Yes	70	71	72.3	141
TNM staging				
I + II	43	29	36.9	72
III + IV	59	64	63.1	123
Distant metastasis				
Yes	13	8	10.8	21
No	89	85	89.2	174

### Immunohistochemistry

Specimens obtained during surgery were quickly fixed in 10% neutral-buffered formalin, dehydrated, and embedded in paraffin using standard techniques. Paraffin-embedded samples were serially sectioned at 4 μm, mounted on adhesion microscope slides, and baked at 60 °C for 1 h. After paraffin sections were deparaffinized in xylene and hydrated through a graded series of alcohol, they were treated in 10 mM (pH 6.0) citrate buffer for 5 min by autoclaving to retrieve antigenicity, cooled to room temperature, blocked with 3% H_2_O_2_ for 10 min, and rinsed 3 times for 3 min in phosphate-buffered saline (PBS, pH 7.2–7.4). The sections were then incubated with 10% normal goat serum in PBS to block nonspecific protein binding, followed by incubation with rabbit anti-human IgG polyclonal anti-Reg IV (Bioss, Beijing, China) at a 1:300 dilution at 37 °C for 2.5 h, and washed with PBS. The Reg IV signals were amplified and visualized with a biotinylated secondary antibody at 37 °C for 20 min and a horseradish peroxidase polymer conjugate at 37 °C for 15 min following the protocol in SP-9000 Histostain™ Plus Kits (ZYMED, South San Francisco, CA, USA). Finally, sections were stained for 5–10 min with 3–3′-Diaminobenzidine (DAB) and counterstained with 0.1% hematoxylin for nuclear staining. A negative control was set simultaneously by replacing the primary antibody with PBS. Two independent investigators analyzed samples, and differences in scoring were discussed until consensus was reached. For evaluating Reg IV expression in the various samples, a scoring criterion was taken from *Sinicrope* et al. [25]. For tumors that showed heterogeneous staining, the predominant pattern was taken into account for scoring. A mean percentage of positive tumor cells was determined in at least 5 areas at × 100 magnification and assigned to one of the 5 following categories: (a) 0, < 5%; (b) 1, 5–25%; (c) 2, 25–50%; (d) 3, 50–75%; and (e) 4, > 75%. The intensity of Reg IV immunostaining was scored as follows: (a) weak, 1+; (b) moderate, 2+; and (c) intense, 3+. The percentage of positive tumor cells and the staining intensity were multiplied to produce a weighted score for each case. Cases with weighted scores of less than 3 were defined as negative; otherwise they were defined as positive. Cytoplasm staining was defined as a positive expression for Reg IV. For SOX9 (1:300, Bioss) staining, the procedure used was as described above. Only nuclear immunoreactivity was considered as a positive expression.

### Total RNA extraction and real-time PCR

Total RNA was extracted by using Trizol reagent (Takara Biotechnology, Dalian, China) and converted to cDNA with a PrimeScript™ RT reagent Kit (Takara Biotechnology), according to the manufacturer’s instructions. Real-time PCR was performed to investigate mRNA expression using a SYBR® Premix Ex Taq™ II kit (Takara Biotechnology). The PCR reaction was performed with following steps: 95 °C for 30 s, and 40 cycles of 95 °C for 5 s to 60 °C for 30 s. Melting curve was obtained at 50 °C to 95 °C, holding 30 s on the 1st step and 5 s on subsequent steps with fluorescence data collection at 0.1 °C intervals. GAPDH was used as an internal control for normalization and RNase-free dH_2_O in place of template DNA was used as the negative control. All samples were assayed in triplicate independently. The expression levels were calculated relative to GAPDH by the delta-delta Ct method. The primers are listed in Table 2.

**Table 2:** Primers used for the real-time PCR

Gene	Forward primer (5′-3′)	Reverse primer
Reg IV	CAGATCCTGGTCTGGCAAGT	ATTCGTTGCTGCTCCAAGTT
SOX9	GGAGATGAAATCTGTTCTGGGAATG	TTGAAGGTTAACTGCTGGTGTTCTG
GAPDH	CAATGACCCCTTCATTGACC	GACAAGCTTCCCGTTCTCAG

### Overexpression of Reg IV gene in gastric carcinoma cells

The gastric carcinoma cell lines MKN-45 and AGS (Chinese Academy of Sciences Institute’s cell resource center, Shanghai) were cultured in DMEM medium containing 10% fetal bovine serum (FBS) in a humidified incubator with 5% CO_2_ at 37 °C.

The full-length coding sequence (CDS) region of the human Reg IV gene was amplified from cDNA and digested with *KpnI/BamHI*. The restricted DNA products were cloned into a PEGFP-c1 vector. The resulting constructs were confirmed by DNA sequencing. The primers used were as follows:

- Reg IV-*KpnI*-F: 5’-CGGGGTACCATGGCTTCCAGAAGCATG-3’
- Reg IV-*BamHI*-R: 5’-CGCGGATCCCTATGGTCGGTACTTGCA-3’

Cells were seeded in 6-well dishes 24 h before transfection at a density of 1–2 × 10^5^ cells per well. For polyethylenimine (PEI)-mediated transfection cases, the suitable cell confluency was 50%–60% at the time of transfection. Two micrograms of DNA expression vector PEGFP-c1 and 6 μL (1 μg/μL) PEI were each diluted with 250 μL Opti-MEM prior to use. The polyplexes were prepared by adding diluted PEI into diluted DNA vectors using an N/P ratio of 10 and incubated for 20 min at room temperature, then added to the cells. The PEGFP-c1 plasmid with no insertion was used as a negative control. mRNA and protein levels were confirmed by real-time PCR and immunoblotting after 48 h.

### Silencing of the Reg IV gene and SOX9 gene by siRNAs in gastric carcinoma cells

A panel of siRNAs, targeting human Reg IV and SOX9 respectively, were designed and synthesized for silencing SOX9 and Reg IV expression from GenePharma (Suzhou, China). A negative siRNA (GenePharma) targeting neither gene was used as negative control. All siRNA sequences used in this study are listed in Table 3. Twenty-four hours before transfection, cells were seeded in 6-well plates with antibiotic-free medium to reach 1 × 10^5^ cells/well eventually. After removal of the medium, cells were washed once with PBS and transfection was conducted according to the manufacturer’s instructions by adding a mixture, composed of 15 μL Lipofectamine RNAiMAX Reagent (Invitrogen, Carlsbad, CA) and 5 μL siRNA (20 μM) or control siRNA, diluted in 50 μL serum-free Opti-MEM Medium (Gibco, Grand Island, NY, USA), to each well with 1950 μL DMEM medium containing 10% FBS at a final concentration of 50 nM siRNA. After an incubation period of 6 h at 37 °C, the transfection medium containing siRNAs was replaced by fresh medium. 48 h after transfection, and the cells were collected for real-time PCR and western blotting. Transfection rates of 60–70% of the cells were accepted for all the experiments.

**Table 3:** siRNA sequences used in the study

siRNA		Sequence 5′ → 3’
SOX9	1	Sense: GCAGCGACGUCAUCUCCAATT
		Antisense: UUGGAGAUGACGUCGCUGCTT
	2	Sense: GAACAAGCCGCACGUCAAGTT
		Antisense: CUUGACGUGCGGCUUGUUCTT
	3	Sense: GACCUUCGAUGUCAACGAGTT
		Antisense: CUCGUUGACAUCGAAGGUCTT
Reg IV	1	Sense: CAUGCUUCUGGAAGCCAUCTT
		Antisense: GAUGGCUUCCAGAAGCAUGTT
	2	Sense: GCUCAUCUCAGCACAGUGCTT
		Antisense: GCACUGUGCUGAGAUGAGCTT
	3	Sense: CUUCAGGAAGCUGAGGAACTT
		Antisense: GUUCCUCAGCUUCCUGAAGTT
Negative control		Sense: UUCUCCGAACGUGUCACGUTT
		Antisense: ACGUGACACGUUCGGAGAATT

### Western blotting

Proteins from cell lysates were extracted using a Tissue or Cell Total Protein Extraction Kit (Shenggong Biotechnology, Shanghai, China) containing protease and phosphatase inhibitor cocktails. Cell lysates were eliminated by centrifugation at 4 °C for 10 min at 12,000 rpm. Culture medium proteins were precipitated by a trichloroacetic acid (TCA) method [26] and dissolved in 8 M urea solution. Five microgram aliquots of protein was separated electrophoretically on 12.5% SDS-PAGE gels and transferred to a nitrocellulose membrane. Nonspecific binding sites were blocked using a buffer with 5% nonfat dry milk in TBST for 1 h at 37 °C. The membranes were then incubated with appropriate antibodies overnight at 4 °C, washed 3 times with TBST, and incubated with horseradish peroxidase-conjugated anti-rabbit secondary antibody, for 1.5 h at 37 °C. Bands were visualized with enhanced chemiluminescence regents (TransGen Biotech, Beijing, China), according to the manufacturer’s protocol, and scanned. The amount of protein expression was corrected by β-actin (ZSGB-Bio, Beijing, China) in the same sample.

### Invasion and migration assay of tumor cells transfected with PEGFP-Reg IV, siR-Reg IV, siR-SOX9 in vitro

The invasion assay was run using a 24-well Transwell invasion chamber with an 8 μm filter (Corning, USA). Cells were transfected with PEGFP-Reg IV, siR-Reg IV, siR-SOX9 and their corresponding negative control, cultured for 36 h and then starved in DMEM containing 1% fetal calf serum for 12 h. The lower compartment of the chambers was filled with DMEM containing 20% fetal calf serum. Cells were seeded on the upper side of the filter which was coated with Matrigel (Corning) in DMEM containing 1% fetal calf serum at a density of 1 × 10^5^ per well. After incubation for 24 h, cells that did not invade through the pores were removed with a cotton swab and the invasive cells were fixed with methanol for 10 min. After being washed in PBS, the invasive cells were stained with 0.1% crystal violet for 10 min and the number of invasive cells on the lower side of the filter was counted using an inverted phase contrast microscope at × 400. For the migration assay, the procedure was the same as with the invasion assay except that the membrane was not coated. The invasion or migration capacity was determined by counting 5 fields per well, and the experiment was repeated in triplicate.

### Wound healing assay of tumor cells transfected with PEGFP-Reg IV, siR-Reg IV, siR-SOX9 in vitro

An in vitro wound healing assay was performed with a modified method as described [27]. Cells were seeded in 6-well dishes, and transfected with PEGFP-Reg IV, siR-Reg IV, siR-SOX9, and their corresponding negative controls until cell confluency to 50%–60%. After creating a confluent cell monolayer, cells were scraped in a straight and homogeneous line with a standard 200-μL pipette tip and reference points were set using a marker pen. The floating cells and debris were removed by washing the cells twice with PBS and then the cells were covered with DMEM containing 1% fetal calf serum for cell culture. The migration status were recorded every 12 h by phase-contrast microscopy. The experiment was repeated in triplicate.

### Statistical analysis

The IBM SPSS Statistics 19 was used for all the calculations and analysis. The association between factors and the quantified clinicopathologic features of tumors were examined by Chi-squared test. The correlation between Reg IV and SOX9 expression was analyzed using Spearman. One-way ANOVO analysis or student’s t test was used for 3- or 2-group analyses, respectively. In all analyses, a *P* value < 0.05 was considered as statistically significant.

## Results

### Reg IV and SOX9 protein expressed in human gastric cancer

**Fig. 1:**
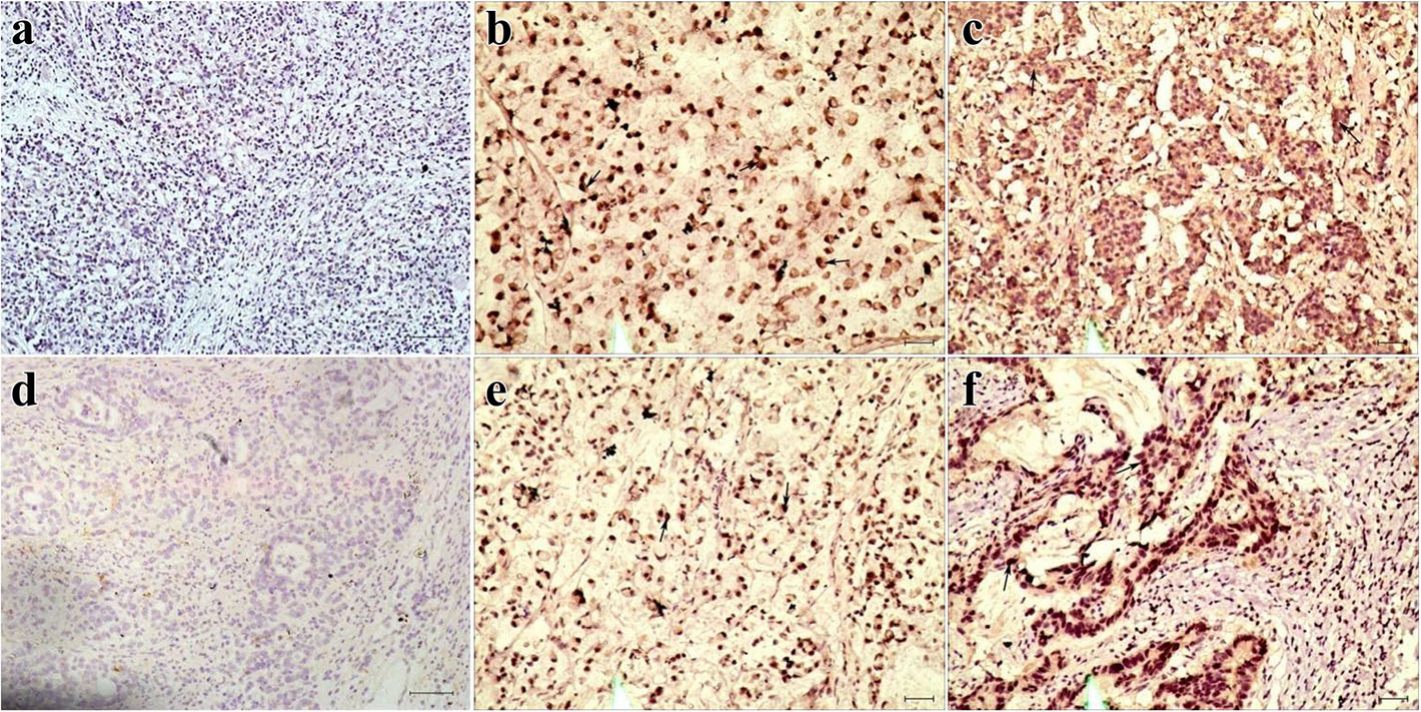
Representative immunohistochemical staining images of Reg IV and SOX9 in gastric cancer tissues. **a** Negative staining of Reg IV; (**b, c)** Cytoplasmic staining of Reg IV; (**d)** Negative staining of SOX9; **(e, f)** Nuclear staining of SOX9. Scale bar =100 μm

We performed IHC analysis to examine Reg IV and SOX9 protein expression in 102 gastric cancer tissues and 40 paired adjacent tissues stained with Reg IV and SOX9 antibody (Fig. 1 and Table 4). The results showed that Reg IV and SOX9 proteins were expressed in both gastric cancer and adjacent tissues (Table 5). Of the 102 gastric tumor cases, Reg IV expression was positive in 55 (53.9%) cancer tissues, which was higher than 13 (32.5%) cases in adjacent tissues (*p* = 0.022). SOX9 expression was significantly higher in 28 (27.5%) cases compared with 2 (5%) cases in adjacent normal tissues (*p* = 0.003). There was a significant correlation between Reg IV protein expression and invasion depth of the tumor (*P* <  0.01). No significant correlations were found between Reg IV protein expression and age, gender, tumor size, tissue differentiation, clinical stage, or lymph node metastasis. SOX9 protein expression showed no significant correlations with age, sex, tumor size, invasion depth of tumor, tissue differentiation, clinical stage, or lymph node metastasis (Table 5).

**Table 4:** The immunohistochemical score of Reg IV and SOX9 in Fig. 1

Parts of Fig. 1	Score (Reg IV)	Parts of Fig. 1	Score (SOX9)
b	12	e	8.4
c	12	f	10.8

**Table 5:** Expression of Reg IV and SOX9 protein and clinicopathologi-cal parameters in gastric cancer tissues

Variable	Reg IV		*P*-Value	SOX9		*P*-Value
	+	–		+	–	
Tumor tissues	55	47	0.022*	28	74	0.003*
Adjacent tissues	13	27		2	38	
Age (year)						
< 60	30	31	0.241	17	44	0.908
≥ 60	25	16		11	30	
Gender						
Man	40	36	0.655	20	56	0.66
Female	15	11		8	18	
Tumor size (cm)						
< 5	32	25	0.613	14	43	0.462
≥ 5	23	22		14	31	
Tissue differentiation						
Moderately-well	33	28	0.965	16	45	0.736
Poorly	22	19		12	29	
Depth of invasion						
T1 + T2	14	30	0	9	18	0.424
T3 + T4	41	17		19	56	
Lymph node metastasis						
No	18	14	0.75	11	21	0.289
Yes	37	33		17	53	
TNM staging						
I + II	23	20	0.94	12	31	0.93
III + IV	32	27		16	43	
Distant metastasis						
Yes	5	8	0.251	4	9	0.93
No	50	39		24	65	

### Reg IV and SOX9 transcript expression in human gastric cancer

The mRNA expression levels of Reg IV and SOX9 were measured by real-time PCR in the 93 cases of gastric cancer and their paired normal tissues. T/N ratios were calculated to represent mRNA expression levels in gastric cancer tissues relative to levels in non-neoplastic tissues: up-regulation (T/*N* >  2), down-regulation (T/*N* < 0.5), and no change in expression (2 > T/*N* > 0.5). The results are shown in Table 6. Reg IV transcription was up-expressed in 40 (43.0%) cases, down-expressed in 36 (38.7%) cases, and did not change in 17 (18.3%) specimens. Up-regulation of SOX9 transcripts was found in 29 (31.2%) of the 93 cases, down-regulation was found in 27 (29.0%) cases and 37 (39.8%) cases had no change in expression. Reg IV transcript expression showed a significant correlation with invasion depth (*p* = 0.005) and lymph node metastasis (*p* < 0.001), and no significant correlations with age, gender, tumor size, tissue differentiation, or clinical stage. SOX9 transcript expression showed a significant correlation with tissue differentiation (*p* = 0.044) and invasion depth (*p* = 0.007). No significant correlations were found between SOX9 transcript expression and age, gender, tumor size, clinical stage, or lymph node metastasis.

**Table 6:** Expression of Reg IV and SOX9 transcripts and correlation between Reg IV and SOX9 and clinicopathological parameters in gastric cancer tissues

Variable	Reg IV			*P* Value	SOX9			*P* Value
	T/N > 2	T/N 2–0.5	T/N < 0.5		T/N > 2	T/N 2–0.5	T/N < 0.5	
Case no.	40	17	36		29	37	27	
Age (year)								
< 60	22	9	17	0.789	14	20	14	0.897
≥ 60	18	8	19		15	17	13	
Gender								
Man	28	15	27	0.344	21	27	22	0.673
Female	12	2	9		8	10	5	
Tumor size (cm)								
< 5	28	8	22	0.257	13	25	20	0.055
≥ 5	12	9	14		16	12	7	
Tissue differentiation								
Moderately-well	26	7	24	0.168	20	17	20	0.044
Poorly	14	10	12		9	20	7	
Depth of invasion								
T1 + T2	8	8	20	0.005	10	21	5	0.007
T3 + T4	32	9	16		19	16	22	
Lymph node metastasis								
No	7	5	25	0.000	13	18	6	0.082
Yes	33	12	11		16	19	21	
TNM staging								
I + II	11	7	11	0.591	8	12	9	0.878
III + IV	29	10	25		21	25	18	

### Association of Reg IV protein expression and SOX9 protein expression

The association of Reg IV protein expression and SOX9 protein is shown in Table 7. The level of Reg IV protein expression showed a positive correlation with SOX9 protein expression (*r* = 0.392, *p* < 0.001).

**Table 7:** Association of Reg IV protein expression with SOX9 protein expression

SOX9	Reg IV			r	*P*-value
	Positive	Negative	Total no.		
Positive	24	4	28	0.392	0.000
Negative	31	43	74		
Total no.	55	47	102		

### Association of Reg IV transcript expression and SOX9 transcript expression

The association between Reg IV transcript expression and SOX9 transcript expression is shown in Table 8. The Reg IV transcript expression levels had a positive correlation with SOX9 transcript expression (*r* = 0.273, *p* = 0.008).

**Table 8:** Association of Reg IV transcript expression with SOX9 transcript expression

SOX9	Reg IV					
	Up-regulation	No difference	Down-regulation	Total no.	r	*P*-value
Up-regulation	16	5	8	29		
No difference	17	9	11	37	0.273	0.008
Down-regulation	7	3	17	27		
Total no.	40	17	36	93		

### Ectopic expression of Reg IV promote tumor cell invasion and migration in vitro

**Fig. 2:**
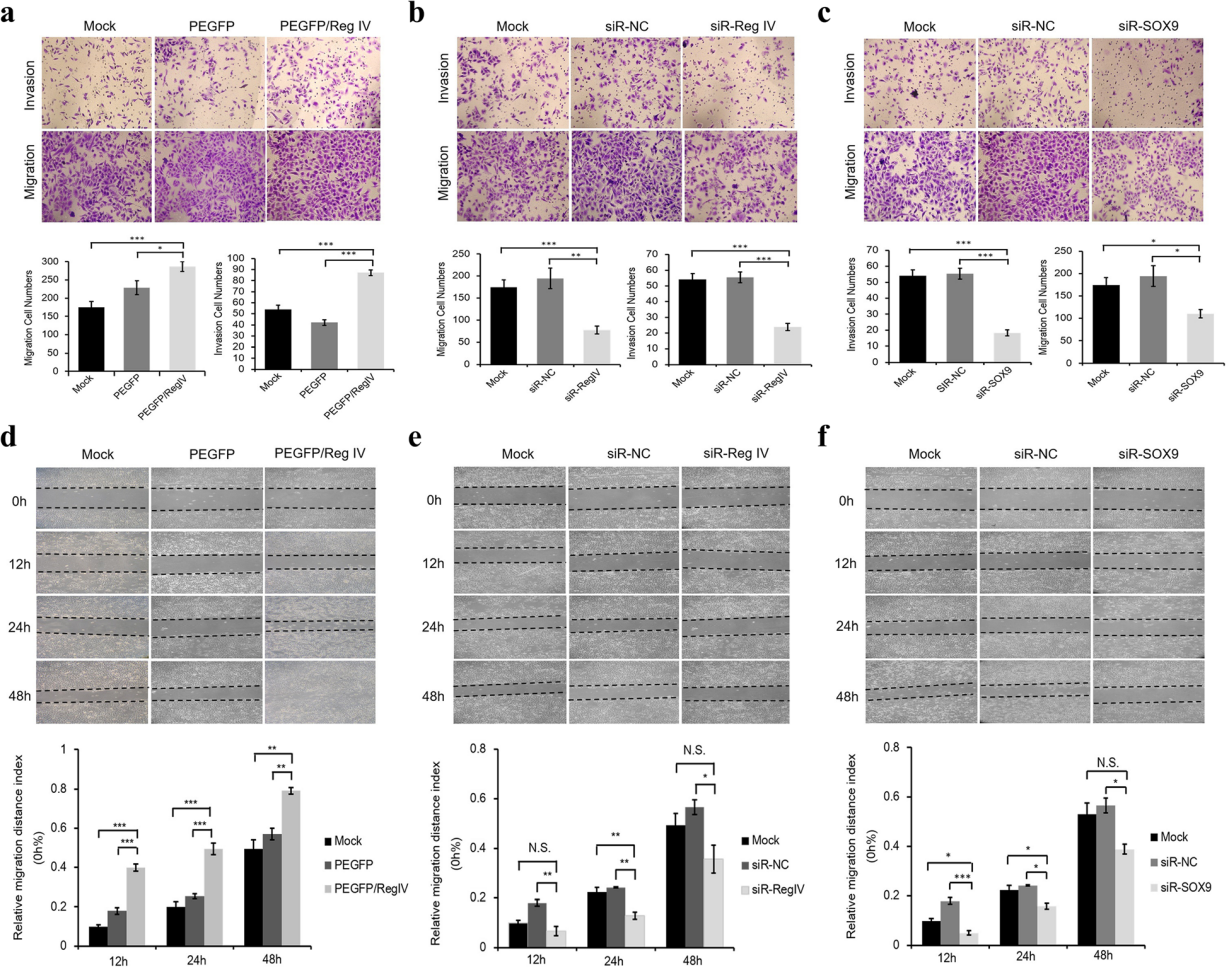
Effects of Reg IV and SOX9 on the invasion and migration abilities in MKN-45 cells. **a** Reg IV overexpression vectors, control empty vectors (PEGFP) and transfection reagents control (Mock) were added to the media. Cells were seeded on the 8 μm-filter chambers after 48 h, and harvested after 24 h. The invasive cells and migrated cells were photographed (top) and counted (bottom); (**b**) effects of Reg IV siRNA (siR-Reg IV) on invasion and migration of MKN-45 cells compared with Mock and control scramble siRNA (siR-NC); (**d**) in wound healing assay, cells were transfected with Reg IV overexpression vectors, control empty vectors (PEGFP), and transfection reagents control (Mock). After creating a confluent cell monolayer, cells were scraped in a straight and homogeneous line. The migration status were recorded every 12 h (top) and calculated (bottom); (**e**) effects of siR-Reg IV on migration of MKN-45 cells compared with Mock and control scramble siRNA; (**f**) effects of siR-SOX9 on migration of MKN-45 cells compared with Mock and control scramble siRNA. The results are shown as Mean ± SEM of 3 independent experiments. ^*^ *P* < 0.05, ^**^ *P* < 0.01, ^***^ *P* < 0.001, N.S. = not significant

**Fig. 3:**
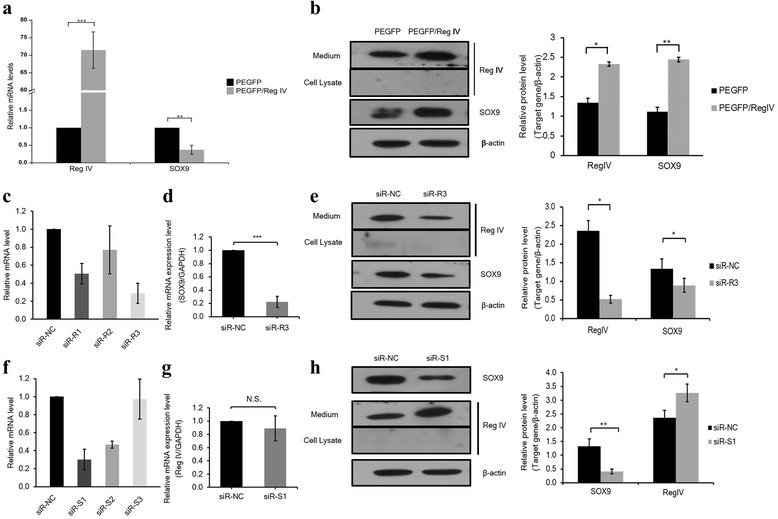
Regulatory relationship of Reg IV and SOX9 in MKN-45 cells. **a** Reg IV overexpression vectors (PEGFP/Reg IV) and control empty vectors (PEGFP) were added to the cells. Cells were harvested after 48 h, total RNA were extracted and converted to cDNA. Real-time PCR was performed to examine the mRNA level of Reg IV and SOX9 in PEGFP/Reg IV and PEGFP treated cells. Relative expression values of mRNA levels were normalized by GAPDH mRNA expression; (**b**) after transfection with PEGFP/Reg IV and PEGFP, the cells and medium were collected. Western blot analysis was done using anti-Reg IV antibody and SOX9 antibody, and bands were visualized (left) and the gray intensity was analyzed (right). β-actin expression level was used as an internal control; (**c**) compared with control scramble siRNA (siR-NC), the mRNA levels of three Reg IV siRNAs (siR-R1, siR-R2, siR-R3) were examined by real-time PCR and siR-R3 showed higher silencing efficiency. **d** the mRNA level of SOX9 was examined in siR-R3-treated cells and the negative control (siR-NC) by real-time PCR; (**e**) after transfection with siR-NC and siR-R3, western blot analysis was done using anti-Reg IV antibody and SOX9 antibody, and bands were visualized (left) and the gray intensity was analyzed (right); (**f**) compared with control scramble siRNA (siR-NC), the mRNA levels of threeSOX9 siRNAs (siR-S1, siR-S2, siR-S3) were examined by real-time PCR and siR-S1 showed higher silencing efficiency; (**g**) the mRNA level of Reg IV was examined in siR-S1-treated cells and the negative control (siR-NC) by real-time PCR; (**h**) After transfection with siR-NC and siR-S1, western blot analysis was done, and bands were visualized (left) and the gray intensity was analyzed (right). The results are shown as Mean ± SEM, *n* = 3. ^*^ *P* < 0.05, ^**^ *P* < 0.01, ^***^ *P* < 0.001, N.S. = not significant

To study the role of Reg IV in human gastric cancer, the PEGFP/Reg IV or control vector were transfected into MKN-45 cells, and the Reg IV mRNA and protein expression levels were measured by real-time PCR and western blot. The invasion and migration ability of the cells transfected with PEGFP/Reg IV or control vector were measured by matrigel transwell assay, no matrigel transwell assay, and wound healing assay. The invasion and migration abilities of the cells transfected with PEGFP/Reg IV were demonstrated to be significantly increased compared with the control (Fig. 2a, d, *p* < 0.05). Increased Reg IV also resulted in increased invasion and migration of AGS cells (see Additional file 1). To confirm the effect of silencing the Reg IV gene to the invasion and migration abilities of tumor cells, silencing effects of 3 pre-designed Reg IV siRNAs were examined in MKN-45 cells, and a scrambled siRNA was used as the negative control. The results showed that the siR-R3 siRNA had a more significant silencing effect and knocked down 71.2% of the Reg IV mRNA in comparison with the scrambled siRNA (Fig. 3c). Therefore this siRNA was selected for silencing the Reg IV gene. The results of the transwell assay and the wound healing assay demonstrated that the invasion and migration abilities of the cells transfected with siR-Reg IV were significantly inhibited compared with the control (Fig. 2b, e, *p<* 0.05). Consistently, Reg IV-knockdown AGS cells also had decreased invasion and migration abilities compared to control cells (see Additional file 1).

### Silencing SOX9 gene inhibits tumor cell invasion and migration in vitro

To study the invasion and migration abilities of tumor cells after silencing the SOX9 gene, silencing effects of 3 pre-designed SOX9 siRNAs were examined in MKN-45 cells, and a scrambled siRNA was used as the negative control. The siR-S1 siRNA showed a more significant silencing effect and knocked down 69.9% of the SOX9 mRNA in comparison with the scrambled siRNA (Fig. 3f). Therefore this siRNA was selected for silencing the SOX9 gene. The results of the transwell assay and the wound healing assay demonstrated that the invasion and migration abilities of the cells transfected with siR-SOX9 were significantly inhibited compared with the control (Fig. 2c, f, *p*< 0.05). SOX9-silenced AGS cells also showed significantly decreased invasion and migration abilities (see Additional file 1).

### Reg IV positively regulates the expression of SOX9 in gastric carcinoma cell lines

To study the potential regulatory relationship between Reg IV and SOX9, the PEGFP/Reg IV or a control vector were transfected into MKN-45 cells, and the SOX9 mRNA and protein expression levels were measured by real-time PCR and western blot. The results demonstrated that the protein expression level of SOX9 in Reg IV overexpressed cells was significantly increased compared with the control (Fig. 3b, *p*< 0.01), while the transcript expression level of SOX9 in Reg IV overexpressed cells was significantly decreased compared with the control (Fig. 3a, *p*< 0.01). The Reg IV gene was silenced by transfecting a siRNA of Reg IV in MKN-45 cells, and the protein and mRNA levels of SOX9 were measured by real-time PCR and western blot. The results demonstrated that the protein and mRNA levels of SOX9 in Reg IV gene silenced cells were significantly decreased compared with the control (Fig. 3d, e, *p*< 0.05). The results of Reg IV gene overexpression and silencing assays demonstrated that SOX9 expression was positively regulated by Reg IV in MKN-45 cells. To determine whether the SOX9 would regulate Reg IV expression in MKN-45 cells, the SOX9 gene was silenced by SOX9 siRNA in MKN-45 cells, and the protein and mRNA levels of Reg IV were measured by real-time PCR and western blot. The results demonstrated that the protein levels of Reg IV in SOX9 gene silenced cells were significantly increased compared with the control (Fig. 3h, *p*< 0.05), but mRNA levels of Reg IV did not significantly change compared with the control (Fig. 3g, *p*> 0.05). The results of silencing the SOX9 gene demonstrated that SOX9 expression inhibition would promote Reg IV protein expression in MKN-45 cells. Similar results were observed in AGS cells (see Additional file 2).

## Discussion

Although advantageous diagnostic and therapeutic strategies have been applied to clinical treatment, gastric cancer is still one of the leading contributors to human cancer death, and the overall prognosis is poor due to its high metastatic potential and relapse rate [2, 28]. Invasion is the initiating process of metastasis and is partly attributable to aberrant gene expression during physiological processes. Previous studies have reported that Reg IV was overexpressed in human gastric cancer, and it may be a promising serum biomarker for gastric cancer, or of peritoneal dissemination of gastric cancer [7, 8, 18, 29]. The results of previous studies demonstrated that ectopic expression of Reg IV is involved in the process of metastasis and progression of gastric cancer. However, the functional mechanism of Reg IV in gastric cancer is poorly understood and needs to be clarified urgently.

In this study, our results demonstrate the following. First, the Reg IV protein expression level was significantly up-regulated in gastric cancer, significantly correlated with the invasion depth of the tumor, and did not significantly correlate with age, sex, tumor size, tissue differentiation, clinical stage, or lymph node metastasis. The expression level of the Reg IV transcript had a significant correlation with the invasion depth of the tumor and lymph node metastasis, and no significant correlation with age, sex, tumor size, tissue differentiation or clinical stage. These results confirmed those of previous studies [7, 8]. Second, the SOX9 protein expression level was significantly up-regulated in gastric cancer, and had no significant correlation with age, sex, tumor size, tissue differentiation, clinical stage, invasion depth of the tumor, or lymph node metastasis. The expression level of the SOX9 transcript had a significant correlation with the invasion depth of the tumor and tissue differentiation, and no significant correlation with age, sex, tumor size, lymph node metastasis, or clinical stage. Third, Reg IV protein and transcript expression level both had a positive correlation with SOX9 protein and transcript expression in gastric cancer.

SOX9 is a SRY-related gene known for chondrogenesis and the development of the male gonad [20, 21], and more and more studies about the ectopic expression of SOX9 and its influence on the progress of carcinogenesis have been reported in several human cancers. Upregulation of SOX9 has been reported to regulate growth and promote the proliferation, invasion, and migration of tumor cells in lung cancer in vitro [30, 31]. Results of another similar study showed that overexpression of SOX9 may accelerate tumor proliferation and promote oncogenesis in the prostate [32]. However, studies of SOX9 in gastric cancer are rarely reported. The expression pattern of SOX9 in the normal human stomach, intestinal metaplasia, and gastric carcinoma have been reported, and the results indicated that SOX9 expression may be associated with gastric carcinogenesis and predict the risk of human intestinal gastric cancer [33]. Further studies revealed that overexpression of SOX9 is related to the progression of gastric cancer [34], and the level of SOX9 methylation increases during progression and decreases in the tumorigenesis of gastric cancer [35]. Fourth, the Reg IV overexpression could significantly increase the invasion and migration abilities of gastric cancer cells, and the Reg IV siRNA and SOX9 siRNA could both significantly inhibit the invasion and migration abilities of gastric cancer cells. Therefore, Reg IV and SOX9 participated in the regulation of tumor invasion and metastasis in gastric cancer. The results above for Reg IV agree with the results of previous studies [19]. Fifth, overexpression of Reg IV significantly promoted SOX9 protein expression in both gastric cancer cells. Reg IV siRNA significantly inhibited SOX9 expression in both gastric cancer cells. SOX9 siRNA significantly promoted Reg IV protein expression in both gastric cancer cells. These findings were similar to the results of a previous study in the MKN-28 and MKN-74 gastric cancer cell line [24]. Consequently, SOX9 may be a downstream gene of Reg IV and is positively regulated by Reg IV. Reg IV may regulate the SOX9 expression and participate in the invasion and metastasis of gastric cancer. The SOX9 down-regulation may promote Reg IV protein expression. Accordingly, there may be feedback efficiency between Reg IV and SOX9. In addition, the phenomenon that the SOX9 protein was upregulated and SOX9 mRNA was down-regulated when Reg IV was overexpressed in MKN-45 cells may be explained as follows. Changes in concentration of a protein depend on the mRNA concentration, translation efficiency and degradation of the existing protein. On average, multi-cellular organisms display the lowest correlations between protein and mRNA concentration. It is therefore possible that the inconsistency is explained by post-transcriptional and post-translation regulation [36]. The regulatory mechanism involving Reg IV and SOX9 in gastric cancer needs to be further investigated.

## Conclusions

Reg IV and SOX9 are both overexpressed and Reg IV expression has a positive correlation with SOX9 expression in gastric cancer. Reg IV expression may promote tumor invasion and metastasis by regulating SOX9, and better understanding the regulatory mechanism of Reg IV may contribute to the identification of new targets for the diagnosis and treatment of patients with gastric cancer.

## Additional files

Additional file 1: **Figure S1.** Effects of Reg IV and SOX9 on the invasion and migration abilities in AGS cells. **a)** PEGFP/Reg IV, PEGFP and Mock were added to the media. Cells were seeded on the 8 μm-filter chambers after 48 h, and harvested after 24 h. The invasive cells and migrated cells were counted (bottom); **b)** effects of siR-Reg IV or siR-SOX9 on invasion and migration of AGS cells compared with Mock and siR-NC; **c)** in wound healing assay, cells were transfected with PEGFP/Reg IV, PEGFP and Mock. After creating a confluent cell monolayer, cells were scraped in a straight and homogeneous line. The migration status were recorded every 12 h (top) and calculated (bottom); **d)** effects of siR-Reg IV or siR-SOX9 on migration of AGS cells compared with Mock and siR-NC. The results are shown as Mean ± SD of 3 independent experiments. ^*^ *P* < 0.05, ^**^ *P* < 0.01, ^***^ *P* < 0.001, N.S. = not significant. (DOCX 457 kb)

Additional file 2: **Figure S2.** Regulatory relationship of Reg IV and SOX9 in AGS cells. PEGFP/Reg IV and PEGFP were added to the cells. Cells were harvested after 48 h, total RNA were extracted and converted to cDNA. Real-time PCR (**a**) was performed to examine the mRNA level of Reg IV and SOX9 in PEGFP/Reg IV and PEGFP treated cells, western blot analysis (**b**) was done using anti-Reg IV antibody and SOX9 antibody, and bands were visualized (left) and the gray intensity was analyzed (right); after transfection with siR-NC and siR-R3, the mRNA level of Reg IV and SOX9 were examined by real-time PCR (**c**), and western blot analysis (**d**) was done; after transfection with siR-NC and siR-S1, the mRNA level of SOX9 and Reg IV (**e**) were examined by real-time PCR, and western blot analysis (**f**) was done. The results are shown as Mean ± SD, *n* = 3. ^*^ *P* < 0.05, ^**^ *P* < 0.01, ^***^ *P* < 0.001, N.S. = not significant. (DOCX 131 kb)

## Abbreviations

5-FU: 5-fluorouracil
IHC: Immunohistochemistry
Reg IV: Regenerating islet gene family member 4
SDS-PAGE: Sodium dodecylsulfate polyacrylamide gel electrophoresis
siRNA: Small interference RNA
SOX9: SRY related high-mobility group box 9
SYR: Sex-determining Region on the Y chromosome
