# Supplementary material for: Expression of Reg IV and SOX9 and their correlation in human gastric cancer
Source: BMC Cancer. 2018 Mar 27;18:344. doi: 10.1186/s12885-018-4285-x (PMC5870489; doi:10.1186/s12885-018-4285-x)
Supplement: Supplementary file 1 — Figure S1. Effects of Reg IV and SOX9 on the invasion and migration abilities in AGS cells. a) PEGFP/Reg IV, PEGFP and Mock were added to the media. Cells were seeded on the 8 μm-filter chambers after 48 h, and harvested after 24 h. The invasive cells and migrated cells were counted (bottom); b) effects of siR-Reg IV or siR-SOX9 on invasion and migration of AGS cells compared with Mock and siR-NC; c) in wound healing assay, cells were transfected with PEGFP/Reg IV, PEGFP and Mock. After creating a confluent cell monolayer, cells were scraped in a straight and homogeneous line. The migration status were recorded every 12 h (top) and calculated (bottom); d) effects of siR-Reg IV or siR-SOX9 on migration of AGS cells compared with Mock and siR-NC. The results are shown as Mean ± SD of 3 independent experiments. * P < 0.05, ** P < 0.01, *** P < 0.001, N.S. = not significant. (DOCX 457 kb) [file 12885_2018_4285_MOESM1_ESM.docx]

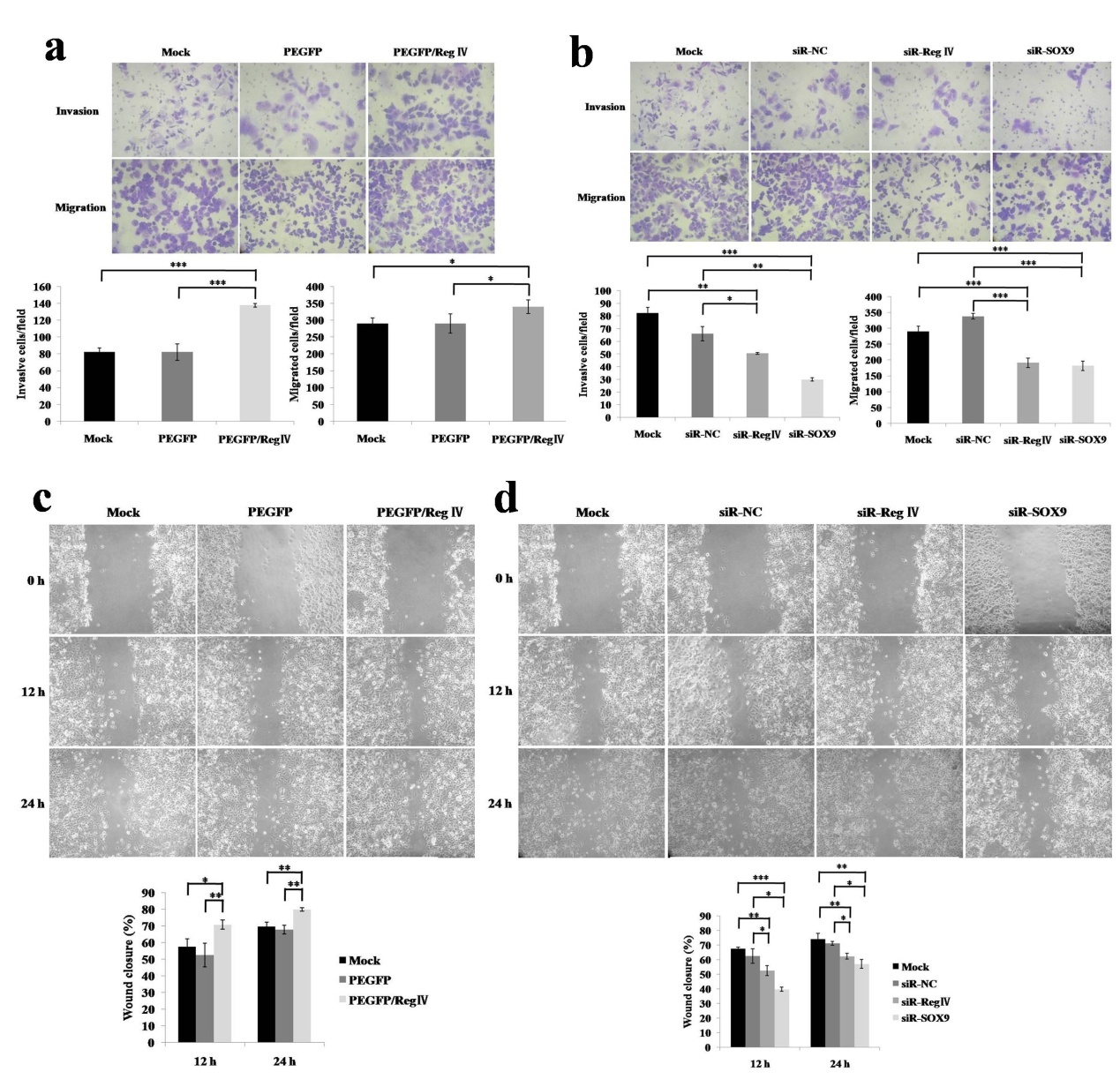


**Fig. S1 Effects of Reg IV and SOX9 on the invasion and migration abilities in AGS cells**. **a)** PEGFP/Reg IV, PEGFP and Mock were added to the media. Cells were seeded on the 8μm-filter chambers after 48 h, and harvested after 24 h. The invasive cells and migrated cells were counted (bottom); **b)** effects of siR-Reg IV or siR-SOX9 on invasion and migration of AGS cells compared with Mock and siR-NC; **c)** in wound healing assay, cells were transfected with PEGFP/Reg IV, PEGFP and Mock. After creating a confluent cell monolayer, cells were scraped in a straight and homogeneous line. The migration status were recorded every 12 h (top) and calculated (bottom); **d)** effects of siR-Reg IV or siR-SOX9 on migration of AGS cells compared with Mock and siR-NC. The results are shown as Mean ± SD of 3 independent experiments. * P < 0.05, ** P<0.01, *** P< 0.001, N.S. = not significant.
