# Supplementary material for: Expression of Reg IV and SOX9 and their correlation in human gastric cancer
Source: BMC Cancer. 2018 Mar 27;18:344. doi: 10.1186/s12885-018-4285-x (PMC5870489; doi:10.1186/s12885-018-4285-x)
Supplement: Supplementary file 2 — Figure S2. Regulatory relationship of Reg IV and SOX9 in AGS cells. PEGFP/Reg IV and PEGFP were added to the cells. Cells were harvested after 48 h, total RNA were extracted and converted to cDNA. Real-time PCR (a) was performed to examine the mRNA level of Reg IV and SOX9 in PEGFP/Reg IV and PEGFP treated cells, western blot analysis (b) was done using anti-Reg IV antibody and SOX9 antibody, and bands were visualized (left) and the gray intensity was analyzed (right); after transfection with siR-NC and siR-R3, the mRNA level of Reg IV and SOX9 were examined by real-time PCR (c), and western blot analysis (d) was done; after transfection with siR-NC and siR-S1, the mRNA level of SOX9 and Reg IV (e) were examined by real-time PCR, and western blot analysis (f) was done. The results are shown as Mean ± SD, n = 3. * P < 0.05, ** P < 0.01, *** P < 0.001, N.S. = not significant. (DOCX 131 kb) [file 12885_2018_4285_MOESM2_ESM.docx]

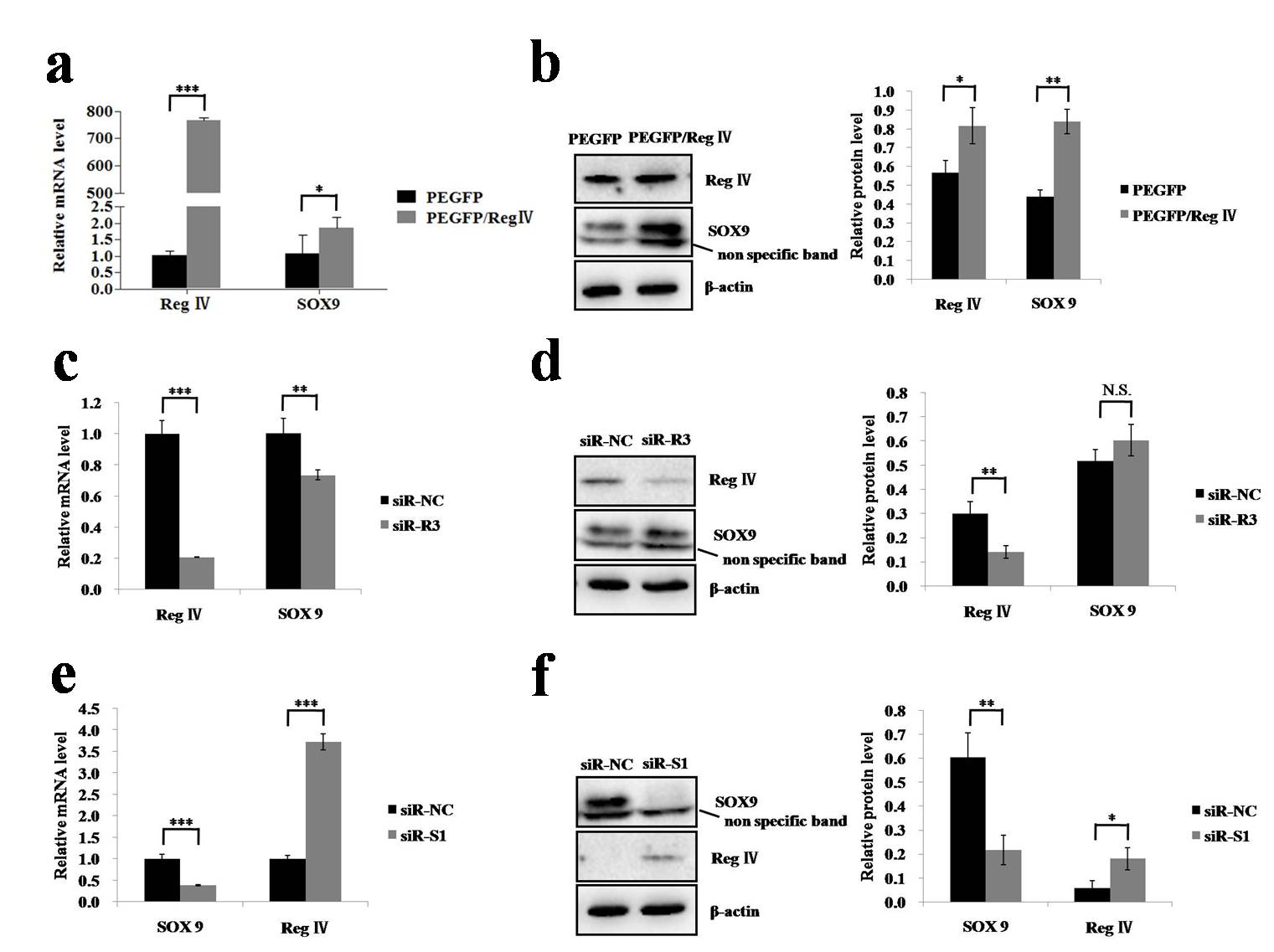


**Fig. S2 Regulatory relationship of Reg IV and SOX9 in AGS cells**. PEGFP/Reg IV and PEGFP were added to the cells. Cells were harvested after 48 h, total RNA were extracted and converted to cDNA. Real-time PCR (a) was performed to examine the mRNA level of Reg IV and SOX9 in PEGFP/Reg IV and PEGFP treated cells, western blot analysis (b) was done using anti-Reg IV antibody and SOX9 antibody, and bands were visualized (left) and the gray intensity was analyzed (right); after transfection with siR-NC and siR-R3, the mRNA level of Reg IV and SOX9 were examined by real-time PCR (c), and western blot analysis (d) was done; after transfection with siR-NC and siR-S1, the mRNA level of SOX9 and Reg IV (e) were examined by real-time PCR, and western blot analysis (f) was done. The results are shown as Mean ± SD, n=3. * P < 0.05, ** P<0.01, *** P<0.001, N.S. = not significant.
